# Long-term PM_2.5_ exposure increases the risk of non-small cell lung cancer (NSCLC) progression by enhancing interleukin-17a (IL-17a)-regulated proliferation and metastasis

**DOI:** 10.18632/aging.103319

**Published:** 2020-06-18

**Authors:** Xie Chao, Liu Yi, Li Lan Lan, Han Yun Wei, Dong Wei

**Affiliations:** 1 Department of Oncology, Shandong Cancer Hospital and Institute, Shandong First Medical University and Shandong Academy of Medical Sciences, Jinan 250117, Shandong Province, P.R. China; 2 Centers of Disease Control and Prevention of Shandong Province, Jinan 250014, Shandong Province, P.R. China; 3 Affiliated Hospital of Binzhou Medical College, Binzhou 256603, Shandong Province, P.R. China; 4 Department of Oncology, Affiliated Hospital of Southwest Medical University, Luzhou 646000, Shihuan Province, P.R. China

**Keywords:** PM2.5, NSCLC, IL-17a, EMT, metastasis

## Abstract

PM_2.5_ is a class of airborne particles and droplets with sustained high levels in many developing countries. Epidemiological studies have indicated that PM_2.5_ is closely associated with the increased morbidity and mortality of lung cancer in the world. Unfortunately, the effects of PM_2.5_ on lung cancer are largely unknown. In the present study, we attempted to explore the role of PM_2.5_ in the etiology of NSCLC. Here, we found that long-term PM_2.5_ exposure led to significant pulmonary injury. Epithelial-mesenchymal transition (EMT) and cancer stem cells (CSC) properties were highly induced by PM_2.5_ exposure. EMT was evidenced by the significant up-regulation of MMP2, MMP9, TGF-β1, α-SMA, Fibronectin and Vimentin. Lung cancer progression was associated with the increased expression of Kras, c-Myc, breast cancer resistance protein BCRP (ABCG2), OCT4, SOX2 and Aldh1a1, but the decreased expression of p53 and PTEN. Importantly, mice with IL-17a knockout (IL-17a^-/-^) showed significantly alleviated lung injury and CSC properties following PM_2.5_ exposure. Also, IL-17a^-/-^-attenuated tumor growth was recovered in PM_2.5_-exposed mice injected with recombinant mouse IL-17a, accompanied with significantly restored lung metastasis. Taken together, these data revealed that PM_2.5_ could promote the progression of lung cancer by enhancing the proliferation and metastasis through IL-17a signaling.

## INTRODUCTION

Particulate matter (PM) is a class of air pollutants, and consists of airborne solid particles and liquid droplets [1]. PM pollution has been one of the highest risk factor among various researched factors in the world [2]. PM_2.5_ is characterized as the particles and droplets with aerodynamic diameter ≤ 2.5 μm [3]. Epidemiological studies have indicated that the increasingly serious air pollution and PM_2.5_ levels are closely associated with health-related issues, including the enhanced hospitalization, increased mortality and mortality because of respiratory problems, shortened lifespan due to long-term PM_2.5_ exposure, as well as the up-regulated incidence and severity of lung cancer [4–6]. Some potential molecular mechanisms have been involved in the progression of lung injury triggered by PM_2.5_ exposure, including excessive oxidative stress, pro-inflammatory response and apoptotic cell death [7–9]. Recently, more and more studies have reported the potential of PM_2.5_ in increasing the risk of lung cancer [10, 11]. In addition, there were reports that demonstrated that the decreased exposure to cigarette smoking attenuated the incidence of lung cancer in the West [4, 7], underlying the importance of air pollution for lung cancer progression. However, little research has focused on the molecular mechanisms. Lung cancer is one of the most common malignancies worldwide and a major reason for tumor-associated death [12, 13]. As reported, NSCLC accounts for approximately 80% of all lung cancer cases, which includes adenocarcinoma and squamous cell carcinoma [14]. The 5-year survival rate of NSCLC is still less than 15%, which is related to the highly malignant potential and the lack of obvious symptoms for early diagnosis [15, 16]. Elevated ability of motility and proliferation were observed in NSCLC cells following PM_2.5_ exposure [17]. In human lung cancer cells, EMT could also be promoted after PM_2.5_ exposure [18]. Furthermore, lung cancer stem cell properties induced by chronic PM_2.5_ exposure has also been reported [19]. Nevertheless, the molecular mechanism by which PM_2.5_ exposure contributes to NSCLC has not been well investigated. It is necessary to thoroughly investigate the mechanisms in NSCLC after exposure to particulate matter to better characterize gene-environment interactions and epigenetic influences on cancer exacerbation.

Interleukin-17 (IL-17) is produced by Th17 cells and other cells, such as CD8^+^ T cells, γδ T cells, mast cells, invariant NKT cells, and granulocytes [20]. IL-17 plays an essential role in regulating inflammatory and autoimmune diseases [21]. Up-regulated IL-17-producing Th17 cells have been indicated in a variety of human carcinoma cases [22, 23]. For instance, the proportion of Th17 cells was elevated within the peripheral blood and tumor tissues of esophageal cancer patients [24]. Tumor-infiltrating Th17 cells were detected in human colorectal cancer, which were associated with shortened disease-free survival [25]. In addition, IL-17 might be implicated in the metastasis of NSCLC through promoting lymphangiogenesis [26]. Recent analysis also indicated that IL-17 might be a critical cytokine involved in the progression of NSCLC. Both *in vivo* and *in vitro* experiments showed that IL-17 could directly enhance the invasion of NSCLC cells [27, 28]. These findings revealed the potential of IL-17 in the promoting the development of NSCLC. IL-17a is one of six members (A-F) of the IL-17 family [29, 30]. In a recent study, the increased expression of IL-17a in the lung of patients with lung adenocarcinoma was reported. Local suppression of IL-17a in the lung of a model with lung cancer showed improved anti-tumor immunity featured by the enhanced IFNγ, a reduced number of T-regulatory cell and the inhibited tumor growth [31]. Although previous studies have illustrated the potential of IL-17a during lung cancer progression, its effects on NSCLC induced by PM_2.5_ have little to be reported.

In this present study, we aimed to further explore the effects of PM_2.5_ on NSCLC progression, as well as its relationship with IL-17a expression change by the *in vivo* and *in vitro* experiments. Here, we showed that long-term PM_2.5_ exposure led to lung injury and CSC properties. IL-17a expression levels were significantly up-regulated by PM_2.5_ in pulmonary tissues, peripheral blood lymphocytes and splenic lymphocytes. NSCLC patients exhibited higher IL-17a expression. In NSCLC cells, PM_2.5_ was discovered to promote the cell proliferation, migration and invasion through up-regulating IL-17a expression levels. Importantly, we for the first time found that IL-17a knockout markedly alleviated PM_2.5_-induced lung injury and CSC properties. Of note, the animal studies showed that PM_2.5_-enhanced tumor growth was clearly abolished by IL-17a knockout in the established tumor xenograft models. Additionally, results by *in vivo* tumor metastasis confirmed that IL-17a knockout inhibited metastasis in PM_2.5_-challenged mice. Therefore, our results demonstrated that PM_2.5_ could elevate the proliferation and metastasis via increasing IL-17a expression levels, accelerating NSCLC progression consequently.

## RESULTS

### PM_2.5_ treatments result in pulmonary injury and fibrosis

**Figure 1:**
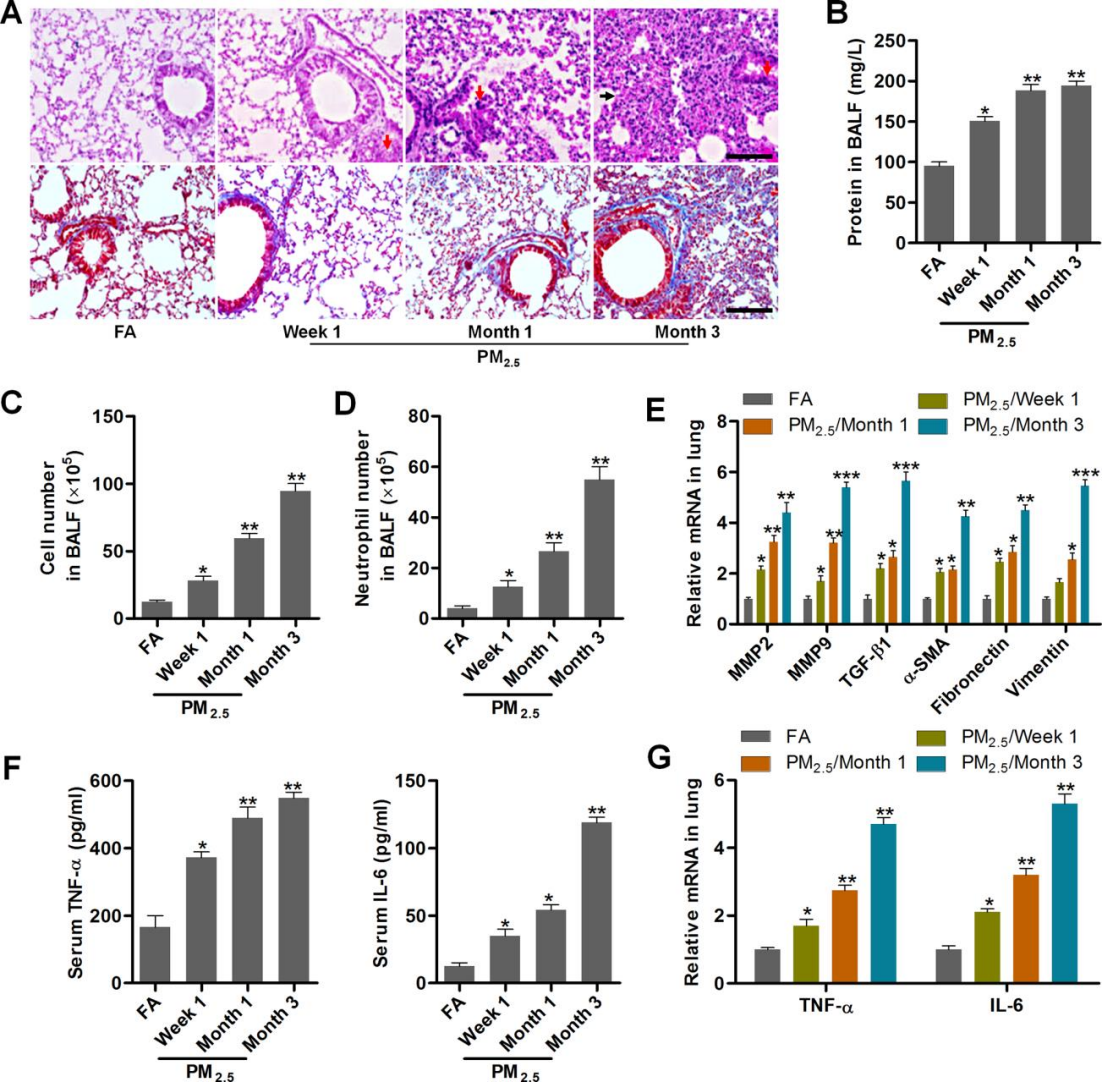
**PM_2.5_ treatments result in pulmonary injury and fibrosis.** (**A**) H&E staining (up panel) and Masson trichrome staining (down panel) of lung sections from mice challenged with PM_2.5_ for the indicated time points (n = 6). Scale bar, 100 μm. (**B**) Protein contents in the BALF were measured in mice treated with PM_2.5_ at the indicated time (n = 8). The number of (**C**) total cells and (**D**) neutrophils of BALF were calculated in PM_2.5_-challenged mice at the indicated time (n = 8). (**E**) RT-qPCR analysis of genes associated with fibrosis, including MMP2, MMP9, TGF-β1, α-SMA, Fibronectin and Vimentin, in lung samples of PM_2.5_-treated mice (n = 4). (**F**) TNF-α and IL-6 levels in serum of PM_2.5_-challenged mice were determined by ELISA (n = 8). (**G**) RT-qPCR analysis was used to assess TNF-α and IL-6 mRNA expression levels in pulmonary samples of mice treated with PM_2.5_ for the indicated time (n = 4). All data are expressed as mean ± SEM. ^*^p<0.05, ^**^p<0.01 and ^***^p<0.001 compared to the FA group.

In order to investigate the effects of PM_2.5_ on NSCLC progression, the pulmonary condition in mice with long-term PM_2.5_ exposure was firstly calculated. Histopathological analysis of lung sections using H&E and Masson staining demonstrated that PM_2.5_ treatment for 3 months caused more severe injury and fibrosis than the FA mice (Figure 1A). Compared to the FA group, PM_2.5_ markedly increased total protein levels in BALF in a time-dependent manner (Figure 1B). Similarly, the number of total cells and neutrophils were significantly increased in mice exposed to PM_2.5_, indicating the critical inflammation in mice (Figure 1C, 1D). EMT is an important process that contributes to fibrogenesis [32]. Then, fibrosis- and/or EMT-associated genes, including MMP2, MMP9, TGF-β1, α-SMA, Fibronectin and Vimentin [33, 34], in lung samples of mice were highly induced by PM_2.5_ administration in a time-dependent manner (Figure 1E). Both serum and lung TNF-α and IL-6 expression levels were greatly up-regulated in PM_2.5_-challenged mice (Figure 1F, 1G). These results showed that long-term PM_2.5_ exposure led to severe pulmonary injury in mice.

### PM_2.5_ treatments lead to cancer stem cell properties in mice

**Figure 2:**
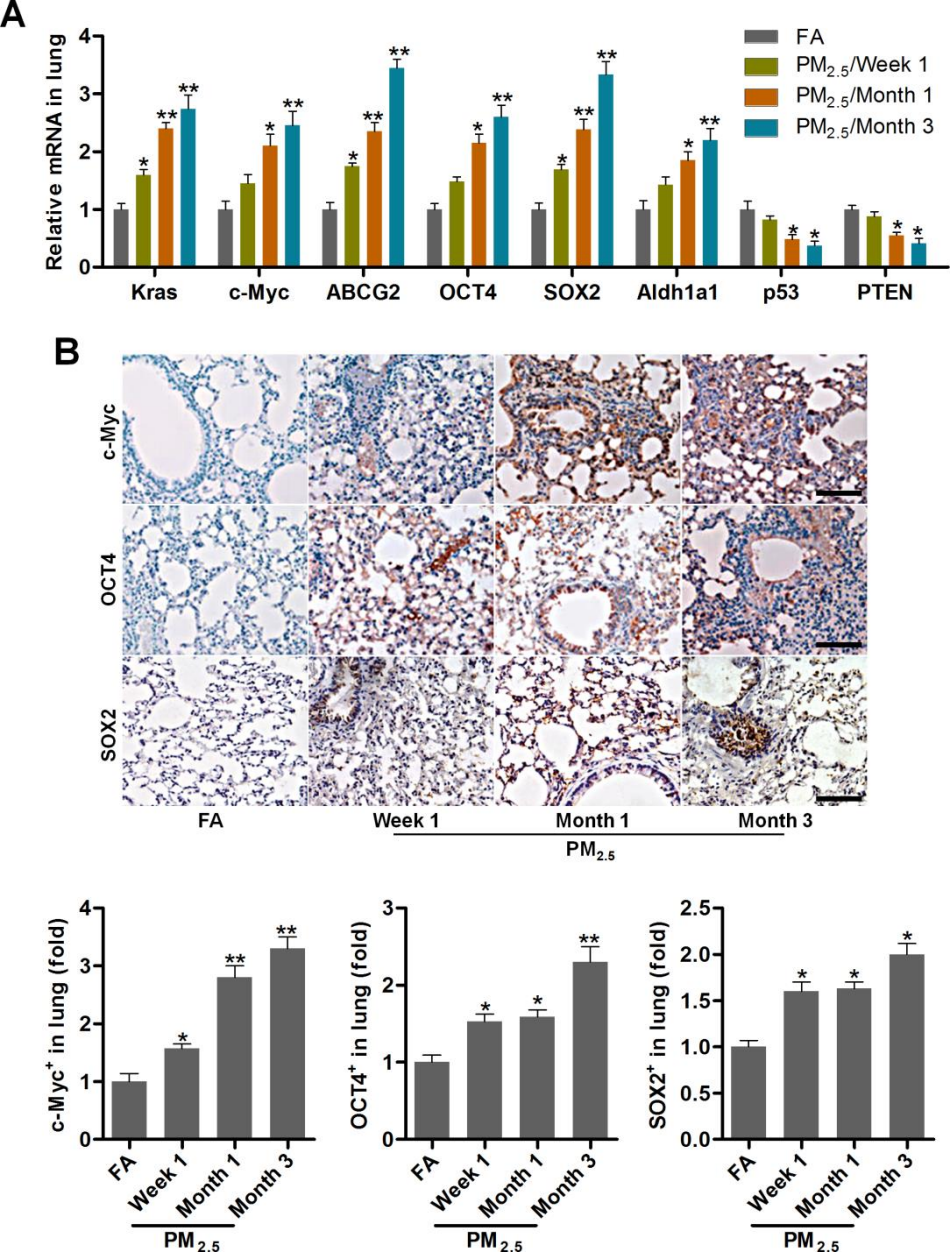
**PM_2.5_ treatments lead to cancer stem cell properties in mice.** (**A**) RT-qPCR analysis was used to measure lung cancer-related biomarkers (Kras, c-Myc, ABCG2, OCT4, SOX2, Aldh1a1, p53 and PTEN) in lung tissues of mice following PM_2.5_ treatment at the indicated time (n = 4). (**B**) IHC staining was performed to calculate c-Myc, OCT4 and SOX2 in pulmonary sections of PM_2.5_-challenged mice (n = 6). The quantification of c-Myc, OCT4 and SOX2 relative expression was exhibited. Scale bar, 100 μm. All data are expressed as mean ± SEM. ^*^p<0.05 and ^**^p<0.01 compared to the FA group.

EMT is a critical event that is often activated during the process of tumor invasion and metastasis. It is also an important and potential driving factor for cancer initiation and development [35]. Then, the lung cancer markers and cancer stem cell features of lung cells (Kras, c-Myc, ABCG2, OCT4, SOX2 and Aldh1a1) [18, 36–38], and the tumor suppressor genes (p53 and PTEN) [39] were calculated using RT-qPCR and/or immunohistochemistry (IHC) staining. As shown in Figure 2A, 2B, Kras, c-Myc, ABCG2, OCT4, SOX2 and Aldh1a1 expression levels were highly induced in pulmonary samples from PM_2.5_-treated mice compared with the FA group. In contrast, PM_2.5_ exposure time-dependently reduced the expression of p53 and PTEN. These results demonstrated that PM_2.5_ long-term exposure could result in the cancer stem cell properties *in vivo*.

### PM_2.5_ enhances IL-17a expression in mice

**Figure 3:**
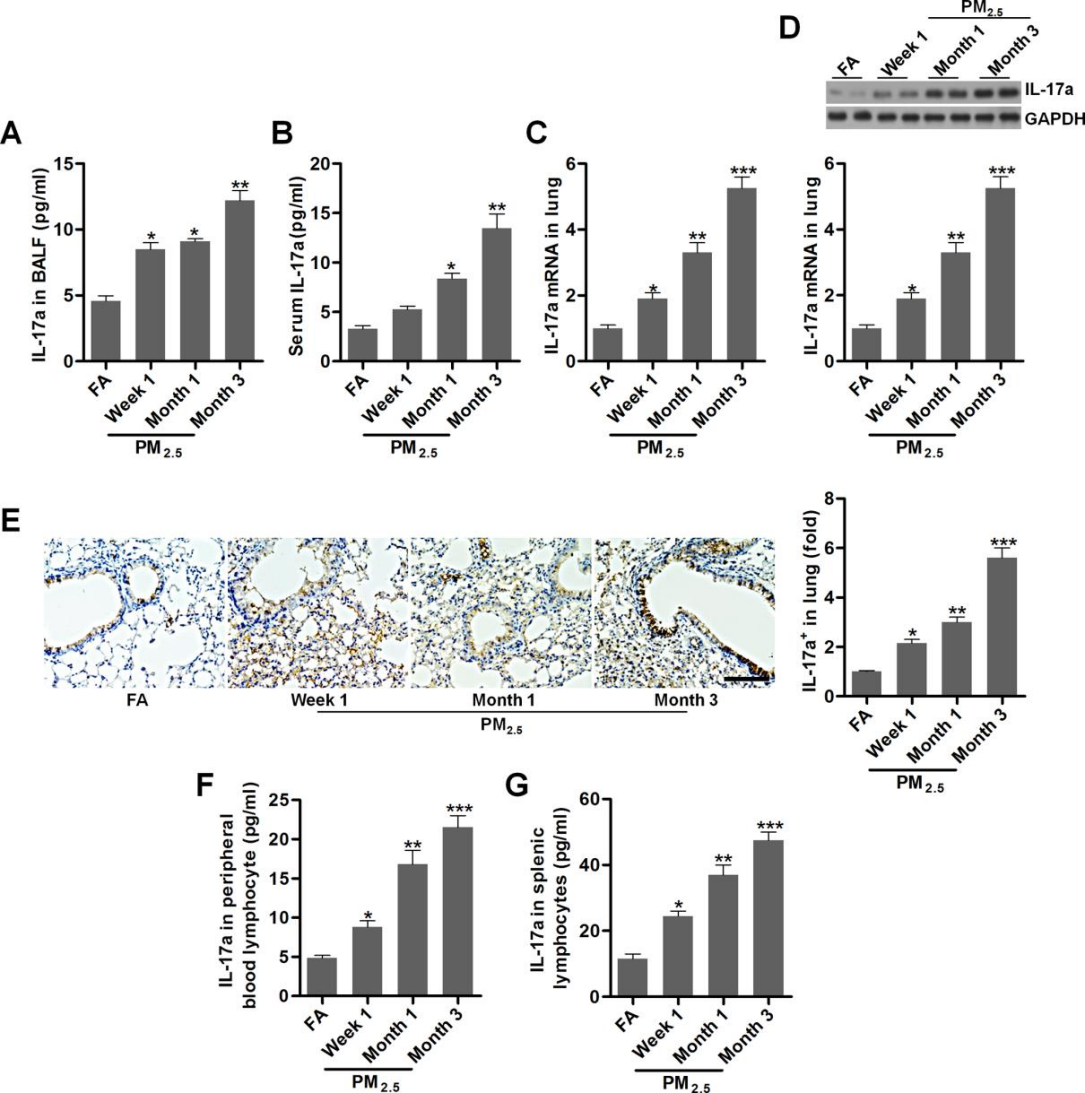
**PM_2.5_ enhances IL-17a expression in mice.** (**A**, **B**) IL-17a contents in BALF and serum were measured by ELISA, respectively (n = 8). (**C**) RT-qPCR, (**D**) western blotting and (**E**) IHC analysis of IL-17a in lung tissues of mice challenged with PM_2.5_ for the indicated time (n = 6). Scale bar, 100 μm. (**F**, **G**) IL-17a levels in the peripheral blood lymphocytes and in splenic lymphocytes were calculated using ELISA analysis (n = 8). All data are expressed as mean ± SEM. ^*^p<0.05, ^**^p<0.01 and ^***^p<0.001 compared to the FA group.

IL-17a has pro-tumor actions, which is associated with angiogenesis [24, 40]. In our study, we found that IL-17a contents in BALF and in serum of mice were markedly increased by PM_2.5_ challenge (Figure 3A, 3B). Higher expression of IL-17a both from mRNA and protein levels were observed in pulmonary samples of mice exposed to PM_2.5_ (Figure 3C–3E). Consistently, ELISA results showed that IL-17a levels in the peripheral blood lymphocytes and in splenic lymphocytes were markedly up-regulated in PM_2.5_-treated mice compared to the FA group (Figure 3F, 3G). Together, findings above indicated that PM_2.5_ exposure could promote IL-17a expression, which might be involved in the initiation and progression of lung cancer.

### IL-17a expression is up-regulated in patients with lung cancer

**Figure 4:**
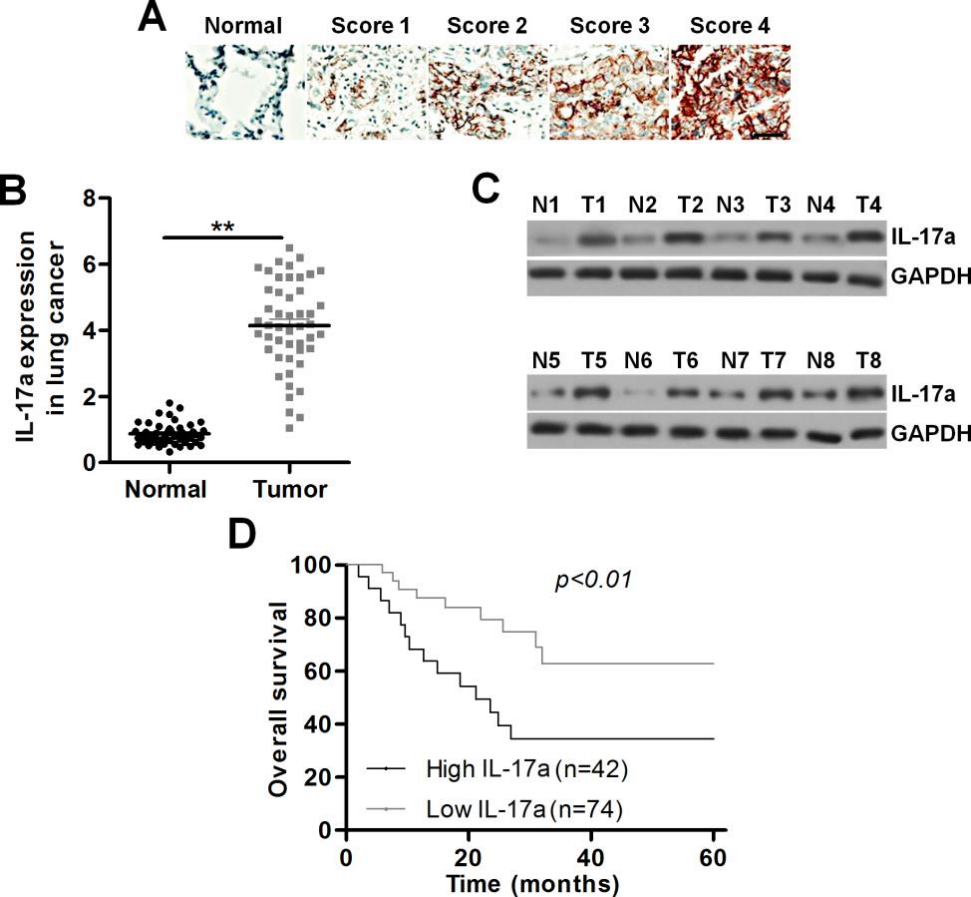
**IL-17a expression is up-regulated in patients with lung cancer.** (**A**) Images showing IL-17a expression levels in tumor samples from patients with lung cancer using IHC. Scale bar, 100 μm. (**B**) IL-17a expression levels in the primary lung tumor specimens and the adjacent normal tissue samples by RT-qPCR (n = 48). (**C**) Western blot analysis of IL-17a expression levels in the primary lung tumor specimens and the paired lung alveolar tissue samples (n = 8). (**D**) Kaplan-Meier survival curves for overall survival (OS) in lung cancer patients according to the expression levels of IL-17a. All data are expressed as mean ± SEM. ^**^p<0.01.

To confirm the regulatory role of IL-17a potentiated by PM_2.5_ during lung cancer development, here the expression change of IL-17a in patients with lung cancer was at first explored. As shown in Figure 4A, an obvious up-regulation of IL-17a in the lung cancer tissues compared to the normal tissues was detected by IHC staining, and IL-17a expression was found to be positively correlated with lung cancer progression. RT-qPCR and western blotting analysis also demonstrated that IL-17a expression levels were higher in lung cancer samples compared to the adjacent normal tissues (Figure 4B, 4C). We also found that patients with low IL-17a expression exhibited better overall survival rates than that of the group with high IL-17a expression (Figure 4D). These results indicated that IL-17a increase was associated with poor clinical outcomes of patients with lung cancer.

### PM_2.5_ elevates the proliferation in NSCLC cells

**Figure 5:**
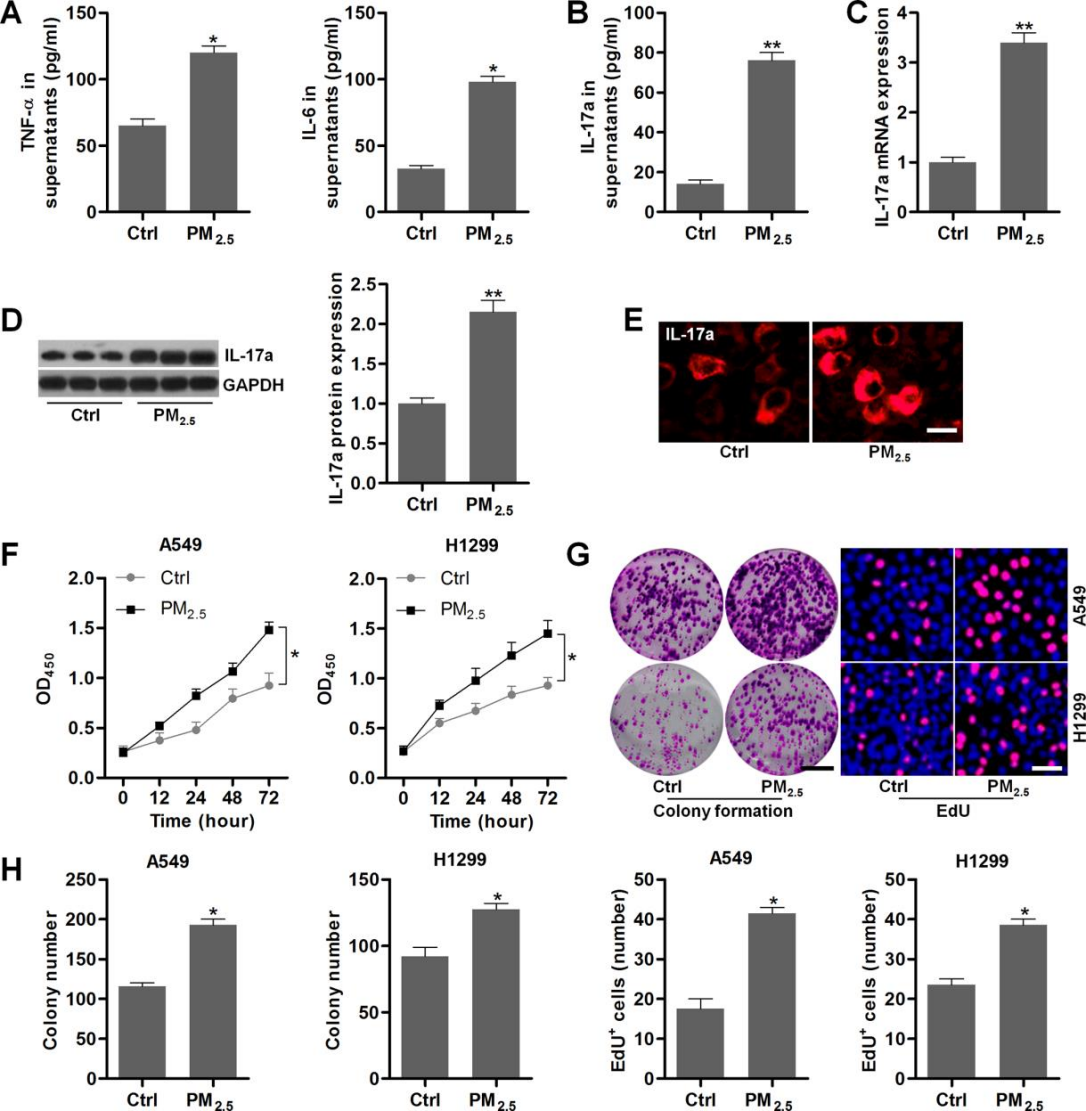
**PM_2.5_ elevates the proliferation in NSCLC cells.** (**A**–**E**) Th17 cells were treated with 100 μg/cm^2^ of PM_2.5_ for 24 h, and then all cells and supernatants were collected for the analysis. (**A**) TNF-α and IL-6 levels in the supernatants were measured using ELISA. (**B**) IL-17a contents in the collected supernatants were calculated using ELISA. (**C**) RT-qPCR and (**D**) western blot analysis were used to measure IL-17a expression levels in the harvested cells. (**E**) IF staining of IL-17a in the harvested cells. Scale bar, 20 μm. (**F**) Th17 cells were treated with 100 μg/cm^2^ of PM_2.5_ for 24 h, and then the conditional medium was collected, and mixed with fresh RPMI1640 absolute medium at 1:3. The composed culture medium was exposed to A549 and H1350 cells for 12, 24, 48 or 72 h. Then, the NSCLC cells were collected for cell proliferation analysis using CCK-8 analysis. (**G**, **H**) Th17 cells were incubated with 100 μg/cm^2^ of PM_2.5_ for 24 h, and then the conditional medium was collected, and mixed with fresh RPMI1640 absolute medium at 1:3. Then, the composed culture medium was subjected to A549 and H1350 cells for another 24 h. Subsequently, all cells were harvested to assess the cell proliferation using colony formation and EdU assays. Scale bar, 100 μm. Quantification of colony formation assay and EdU was exhibited. All data are expressed as mean ± SEM. ^*^p<0.05 and ^**^p<0.01 compared to the Ctrl group.

To confirm the effects of PM_2.5_ on NSCLC, the *in vitro* experiments using A549 and H1299 were performed. At first, we found that PM_2.5_ exposure led to significant up-regulation of TNF-α and IL-6 in the supernatants of Th17 cells compared to the Control group (Figure 5A). Higher IL-17a contents in supernatants collected from PM_2.5_-exposed Th17 cells were observed (Figure 5B). In addition, IL-17a expression levels in Th17 cells were also markedly increased by PM_2.5_ treatment in comparison to the Control group (Figure 5C–5E). Results above showed that PM_2.5_ could up-regulate IL-17a expression levels in Th17 cells.

Then, NSCLC cell lines of A549 and H1299 were incubated in the culture medium composed of the fresh medium and the conditional medium from Th17 cells with or without PM_2.5_ stimulation at 3:1. CCK-8 analysis showed that A549 and H1299 cells cultured in medium from Th17 cells with PM_2.5_ treatment exhibited higher cell proliferative activity (Figure 5F). Similar proliferative trends were observed in A549 and H1299 cells by the colony formation and EdU assays (Figure 5G, 5H). These results demonstrated that PM_2.5_ exposure could promote the proliferation of NSCLC cells.

### PM_2.5_ contributes to the migration and invasion of NSCLC cells

**Figure 6:**
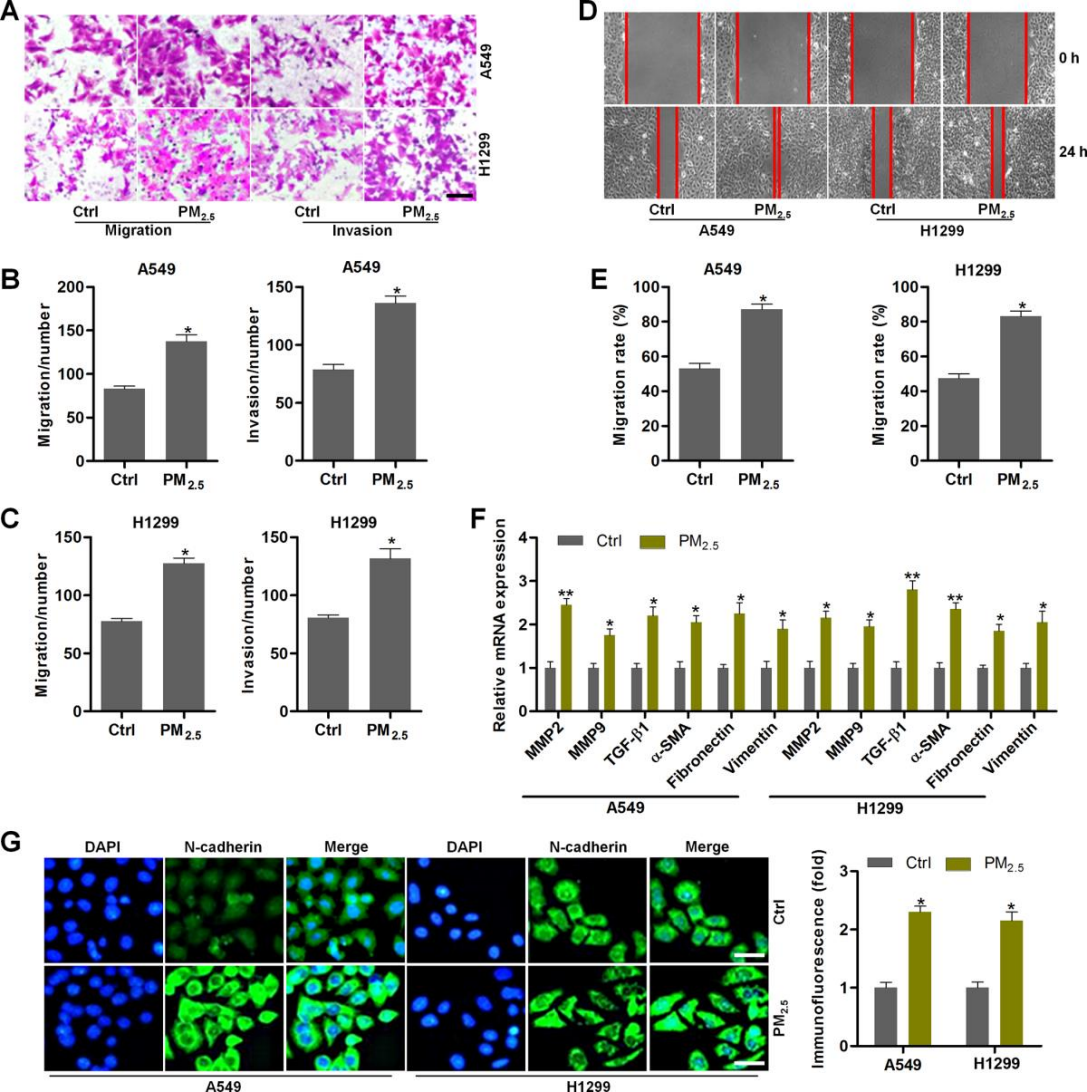
**PM_2.5_ contributes to the migration and invasion of NSCLC cells.** (**A**–**G**) Th17 cells were exposed to 100 μg/cm^2^ of PM_2.5_ for 24 h. Then, the obtained medium was collected, and mixed with fresh RPMI1640 absolute medium at 1:3 dilutions. Next, A549 and H1350 cells were treated with the composed culture medium for another 24 h. Subsequently, all cells were collected for the following analysis. (**A**) Transwell analysis was used to determine the migration and invasion of lung cancer cells. Scale bar, 100 μm. (**B**, **C**) Quantification of the number of cells in migration and invasion. (**D**) Wound healing analysis was performed to assess the migration of lung cancer cells. (**E**) The number of NSCLC cells in migration was quantified following wound healing analysis. (**F**) RT-qPCR analysis was used to calculate the mRNA expression levels of genes associated with EMT. (**G**) IF staining of N-cadherin in A549 and H1350 cells treated as indicated. Scale bar, 50 μm. All data are expressed as mean ± SEM. ^*^p<0.05 and ^**^p<0.01 compared to the Ctrl group.

We next explored the effect of PM_2.5_ exposure on NSCLC cell migration and invasion. A549 and H1299 cells have high migratory and invasive abilities [41]. Under PM_2.5_-exposed circumstances, A549 and H1299 cells had higher migration and invasion compared to the control group by transwell analysis (Figure 6A–6C). Wound healing analysis confirmed that PM_2.5_ exposure enhanced the migration of cells compared with the untreated control cells (Figure 6D, 6E). Moreover, the mRNA expression levels of EMT-associated genes including MMP2, MMP9, TGF-β1, α-SMA, Fibronectin and Vimentin were markedly up-regulated in NSCLC cells cultured in medium containing supernatants from PM_2.5_-incubated Th17 cells (Figure 6F). IF staining further demonstrated that the conditional medium from PM_2.5_-treated Th17 cells led to the expression of N-cadherin in A549 and H1299 cells (Figure 6G). Collectively, the results in this section demonstrated that PM_2.5_-induced circumstances could accelerate EMT in NSCLC.

### IL-17a treatment promotes the proliferation and EMT in NSCLC cells

**Figure 7:**
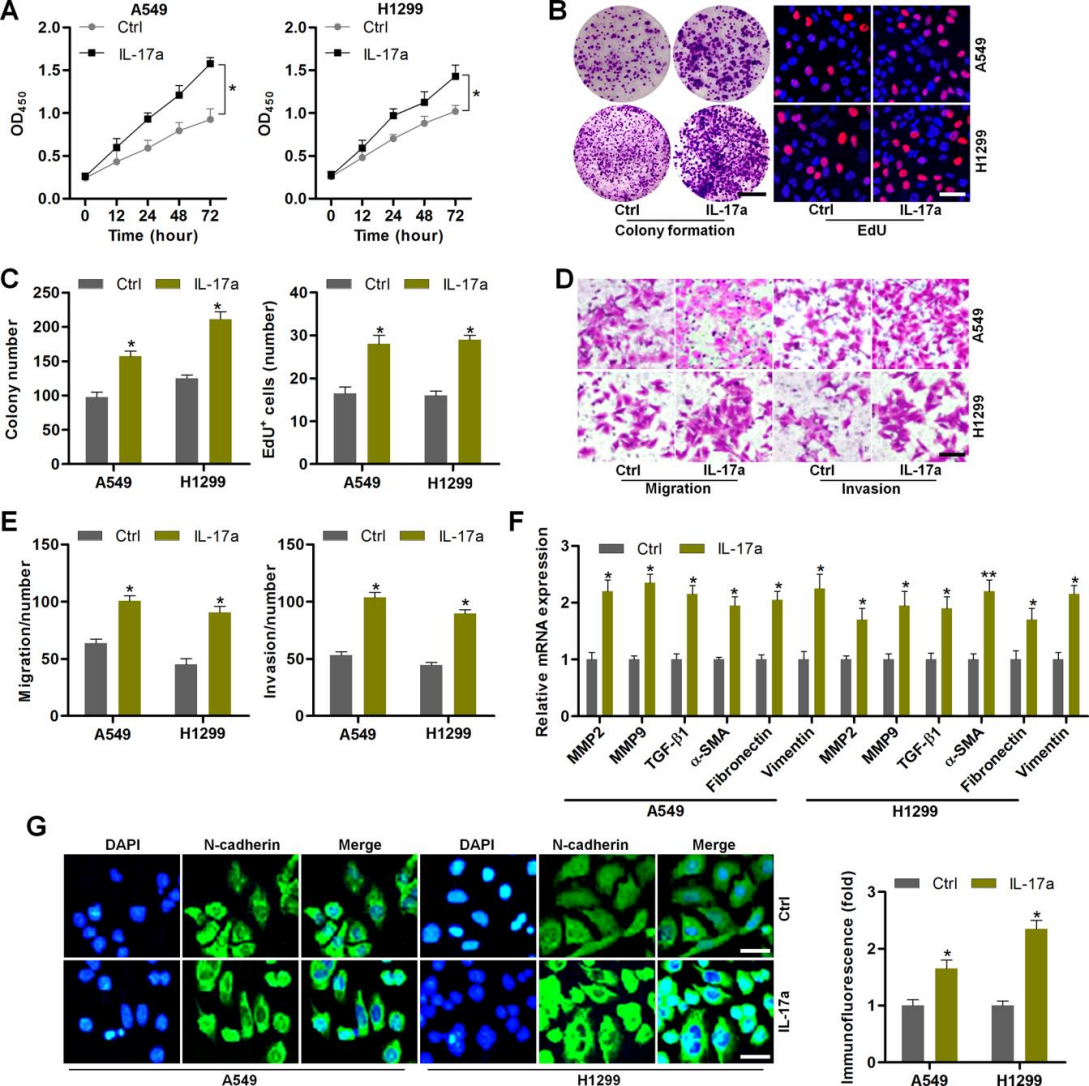
**IL-17a treatment promotes the proliferation and EMT in NSCLC cells.** (**A**–**G**) A549 and H1350 cells were treated with or without IL-17a (100 ng/ml) for 24 h, and then were collected for the following studies. (**A**) CCK-8 analysis was used to determine the cell proliferative activity. (**B**) Colony formation and EdU assays were used to determine the cell proliferative condition. Scale bar, 100 μm. (**C**) Quantification of the number of cells by colony formation and EdU analysis. (**D**) Transwell analysis was used to calculate the number of cells in migration and invasion. Scale bar, 100 μm. (**E**) Quantification of the counts of the migrated and invaded cells. (**F**) RT-qPCR analysis was used to measure EMT-associated genes in A549 and H1350 cells. (**G**) IF staining of N-cadherin in A549 and H1350 cells. Scale bar, 50 μm. All data are expressed as mean ± SEM. ^*^p<0.05 and ^**^p<0.01 compared to the Ctrl group.

To further confirm the effects of IL-17a on NSCLC progression, Recombinant Human IL-17a was then subjected to A549 and H1299 cells. CCK-8 analysis demonstrated that IL-17a treatment markedly promoted the proliferation of NSCLC cells (Figure 7A). Colony formation and EdU assays confirmed the role of IL-17a in promoting NSCLC proliferation (Figure 7B, 7C). Transwell analysis demonstrated that the number of cells in migration and invasion was markedly up-regulated by IL-17a addition (Figure 7D, 7E). Furthermore, MMP2, MMP9, TGF-β1, α-SMA, Fibronectin and Vimentin mRNA levels were significantly induced by IL-17a in A549 and H1299 cells (Figure 7F). The role of IL-17a in promoting N-cadherin was verified by IF staining as displayed in Figure 7G. Together, results in this regard illustrated that IL-17a could enhance the proliferation and EMT in NSCLC.

### IL-17a knockout alleviates pulmonary injury and cancer stem cell properties in mice following PM_2.5_ exposure for 3 months

**Figure 8:**
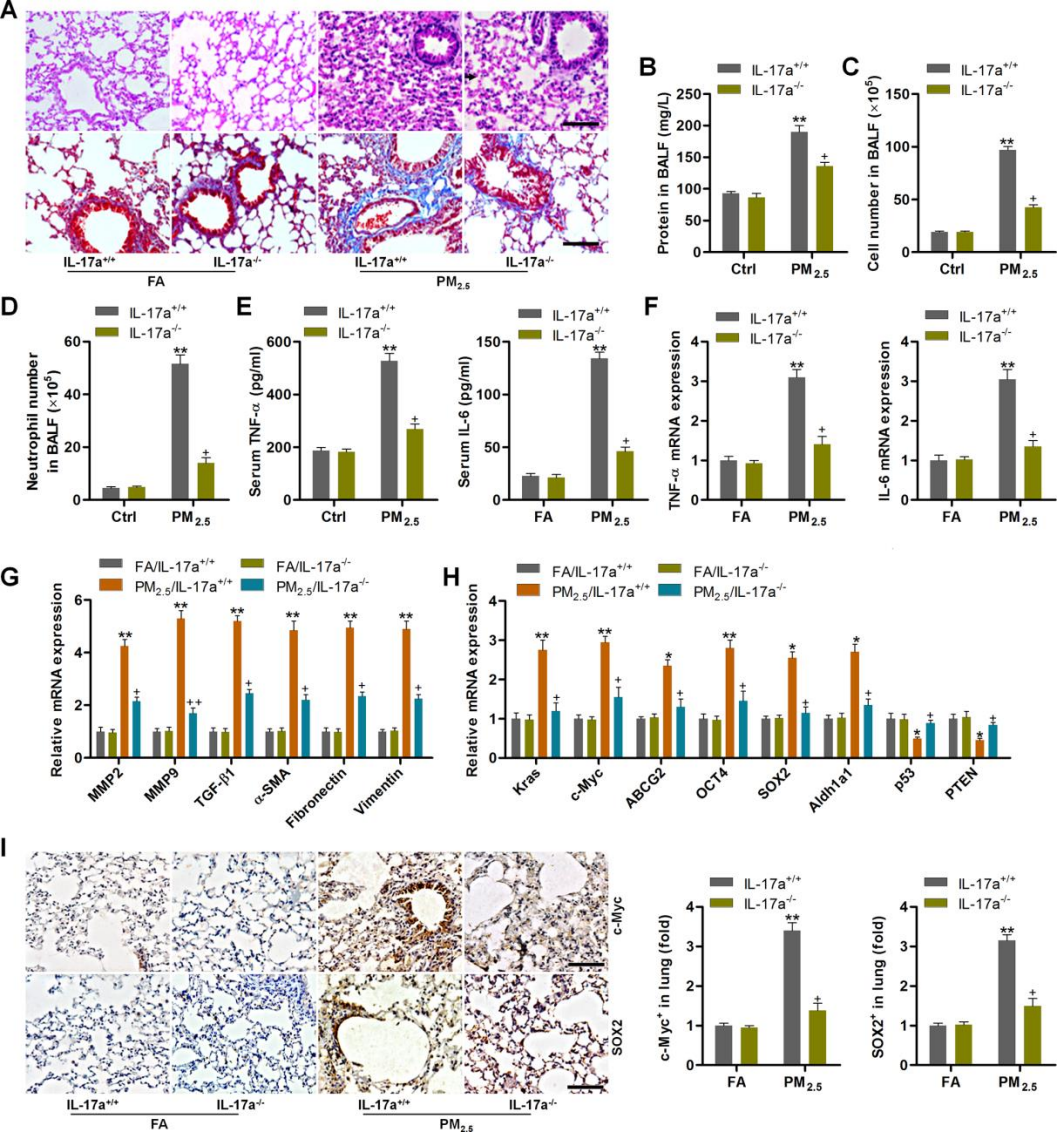
**IL-17a knockout alleviates pulmonary injury and cancer stem cell properties in mice following PM_2.5_ exposure for 3 months.** (**A**) H&E staining (up panel) and Masson trichrome staining (down panel) of lung sections from IL-17a^+/+^ and IL-17a^-/-^ mice challenged with or without PM_2.5_ for 3 months (n = 6). Scale bar, 100 μm. (**B**) Protein levels in BALF were measured (n = 8). (**C**) The total number of cells in BALF was assessed (n = 8). (**D**) The number of neutrophils in BALF was measured (n = 8). (**E**) Serum TNF-α and IL-6 levels in mice were measured by ELISA (n = 8). (**F**) TNF-α and IL-6 mRNA levels in the pulmonary samples were determined using RT-qPCR analysis (n = 4). (**G**) Fibrosis-associated genes as shown were tested using RT-qPCR analysis (n = 4). (**H**) Genes associated with lung cancer progression were calculated using RT-qPCR analysis (n = 4). (**I**) IHC staining of c-Myc and SOX2 in pulmonary sections from the indicated groups of mice (n = 6). Scale bar, 100 μm. All data are expressed as mean ± SEM. ^*^p<0.05 and ^**^p<0.01 compared to the FA/IL-17a^+/+^ group; *^+^p<0.05* compared to the PM_2.5_/IL-17a^+/+^ group.

The *in vivo* and *in vitro* experiments above demonstrated that PM_2.5_ could up-regulate IL-17a expression to subsequently promote the progression of NSCLC via enhancing the proliferation and EMT processes. In order to further confirm the significant role of IL-17a regulated by PM_2.5_ in pulmonary progression and lung cancer development, IL-17a knockout (IL-17a^-/-^) mice were used subsequently. As shown in Figure 8A, H&E and Masson staining showed that PM_2.5_-induced pulmonary injury and fibrosis were attenuated when IL-17a was deleted in mice. Total proteins in BALF induced by long-term PM_2.5_ exposure were significantly abolished in IL-17a^-/-^ mice (Figure 8B). Markedly reduced total number of cells and the number of neutrophils in BALF were detected in IL-17a^-/-^ mice following PM_2.5_ exposure (Figure 8C, 8D). Compared to the PM_2.5_/IL-17a^+/+^ group, TNF-α and IL-6 expression levels in serum and lung tissues were markedly decreased in IL-17a^-/-^ mice after PM_2.5_ exposure for 3 months (Figure 8E, 8F). RT-qPCR analysis showed that IL-17a deletion evidently reduced the expression of MMP2, MMP9, TGF-β1, α-SMA, Fibronectin and Vimentin in pulmonary samples of PM_2.5_-challenged mice compared to that of the wild type mice (Figure 8G). Furthermore, lung cancer markers including Kras, c-Myc, ABCG2, OCT4, SOX2 and Aldh1a1 induced by PM_2.5_ were also markedly abolished when IL-17a was knocked out; however, p53 and PTEN mRNA expression levels restrained by PM_2.5_ were significantly rescued in mice with the loss of IL-17a (Figure 8H). IHC staining confirmed the role of IL-17a^-/-^ in suppressing c-Myc and SOX2 expression in lung tissues of PM_2.5_-exposed mice (Figure 8I). The *in vivo* results above demonstrated that reducing IL-17a expression could alleviate PM_2.5_-induced pulmonary injury and the expression of genes associated with lung cancer progression.

### PM_2.5_-promoted tumor growth and metastasis are associated with IL-17a in xenograft mouse models

**Figure 9:**
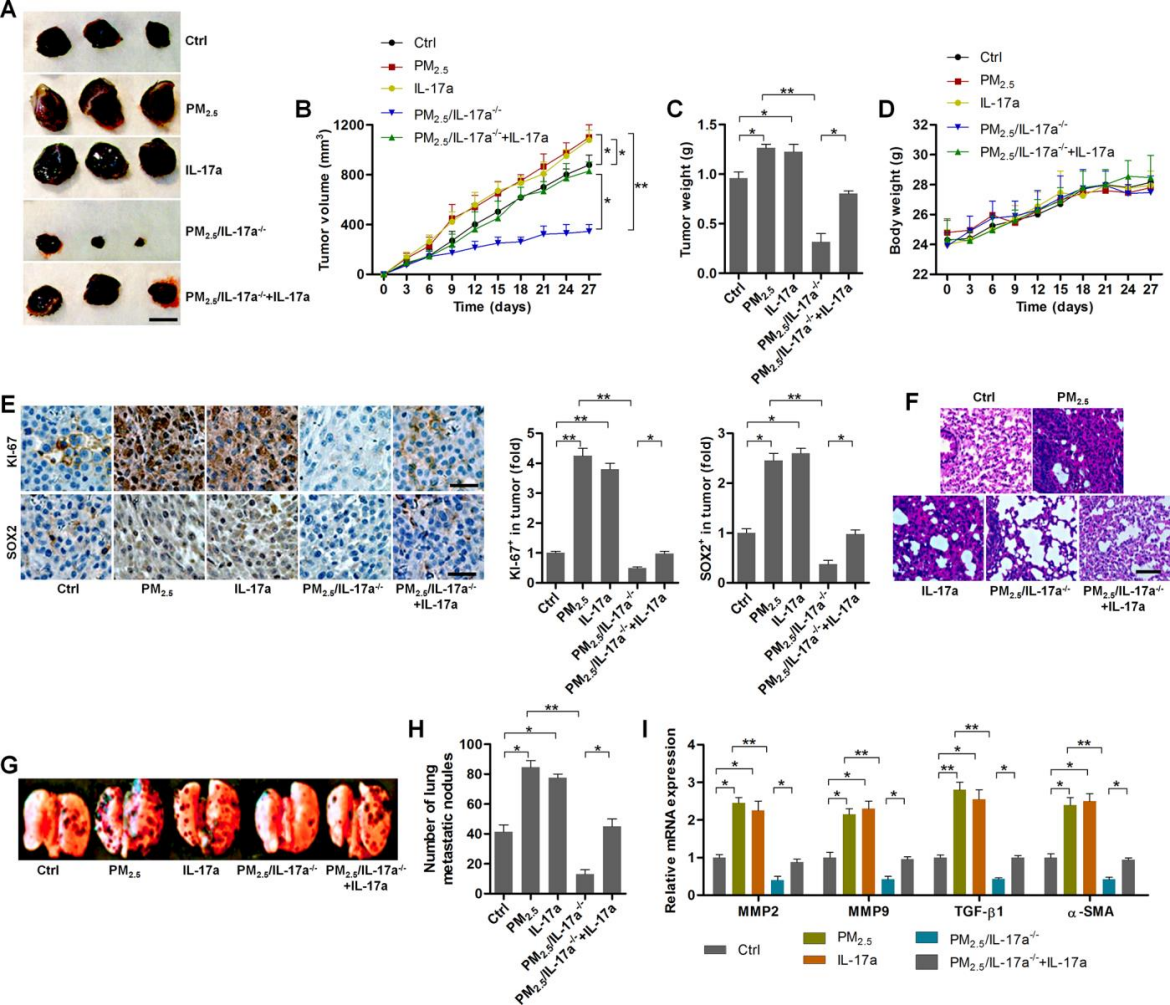
**PM_2.5_-promoted tumor growth and metastasis are associated with IL-17a in xenograft mouse models.** (**A**) Representative images of tumor samples isolated from each group of mice as indicated (n = 6). Scale bar, 1 cm. (**B**) Tumor volume was measured (n = 6). (**C**) Tumor weight was recorded (n = 6). (**D**) The body weight of mice was recorded (n = 6). (**E**) IHC staining was used to determine KI-67 and SOX2 expression levels in the tumor sections (n = 4). Scale bar, 100 μm. (**F**) H&E staining of pulmonary tissues (n = 4; Scale bar, 100 μm) and (**G**) pictures of lung samples isolated from the indicated groups of mice to calculate the metastatic nodules on the surface of lungs (n = 6). (**H**) The number of lung metastatic nodules was quantified (n = 6). (**I**) RT-qPCR analysis was used to calculate the expression of metastasis-associated genes as displayed (n = 4). All data are expressed as mean ± SEM. ^*^p<0.05 and ^**^p<0.01.

In this regard, A549-drived xenograft mouse models were established using C57BL6 mice to further explore the effects of PM_2.5_ on NSCLC progression, as well as the potential of IL-17a involved. As shown in Figure 9A–9C, the tumor size, volume and weight were highly promoted by PM_2.5_ exposure compared to the Ctrl group, and similar results were observed in mice injected with IL-17a. Of note, PM_2.5_-enhanced tumor growth was clearly abrogated in IL-17a^-/-^ mice; however, this effect was restored when IL-17a was again injected to mice. In addition, no significant difference was observed in the change of body weight of mice from different groups (Figure 9D). KI-67, as a marker of cell proliferation, was clearly up-regulated by PM_2.5_ exposure and IL-17a injection when compared to the Ctrl group. IL-17a deficiency considerably reduced KI-67 expression in tumor samples from PM_2.5_-challenged mice, while being restored by IL-17a reinjection. Similar expression alterations were detected in SOX2 by IHC staining (Figure 9E). The *in vivo* metastasis was then investigated. Lung metastasis was greatly promoted in mice treated with PM_2.5_ or IL-17a. Of note, compared to the wild type mice, IL-17a^-/-^ mice exhibited markedly alleviated metastasis *in vivo* following PM_2.5_ exposure, but this effect was abrogated when mice were re-injected with IL-17a (Figure 9F–9H). Finally, RT-qPCR results indicated that metastatic markers, including MMP2, MMP9, TGF-β1 and α-SMA, were markedly up-regulated by PM_2.5_ exposure or IL-17a addition. Consistently, mice with IL-17a deficiency exhibited reduced expression of these genes, which were, however, significantly rescued by IL-17a reinjection to mice (Figure 9I). Collectively, these *in vivo* results illustrated that PM_2.5_ exposure could accelerate the growth of NSCLC and metastasis, which was largely dependent on the expression of IL-17a.

## DISCUSSION

NSCLC is a critical disease with high morbidity and mortality worldwide. Long-term PM_2.5_ exposure is tightly associated with NSCLC progression. Increasing studies have shown the importance of IL-17a in promoting tumor development, including NSCLC, via multiple mechanisms [23–29]. In this research, chronic PM_2.5_ exposure at high dose caused significant pulmonary damage and fibrosis. On the other, the expression levels of lung cancer biomarkers were highly induced by PM_2.5_ exposure in lung tissues. IL-17a expression was markedly up-regulated in pulmonary samples, peripheral blood lymphocytes and splenic lymphocytes from PM_2.5_-exposed mice. Clinical analysis demonstrated that IL-17a expression was significantly up-regulated in NSCLC patients. PM_2.5_ exposure caused IL-17a expression in the isolated Th17 cells. Essentially, NSCLC cells cultured in the conditional medium from PM_2.5_-incubated Th17 cells exhibited markedly increased cell proliferation and EMT processes. Consistently, recombinant human IL-17a considerably led to NSCLC cell proliferation, migration and invasion. Notably, the *in vivo* experiments further demonstrated that PM_2.5_-induced pulmonary damage was alleviated in mice with IL-17a knockout. In addition, IL-17a deletion markedly abolished the role of PM_2.5_ in promoting tumor growth and metastasis in mouse models. Taken together, all these results expanded the understanding of pulmonary carcinogenesis of PM_2.5_ through increasing IL-17a expression levels, and thus IL-17a could be a biomarker for lung cancer prediction under the stress of PM_2.5_.

Several molecular mechanisms contribute to the onset and pathogenesis of PM_2.5_-induced lung injury, such as oxidative stress, inflammation and fibrosis [7–9]. In our present study, we further confirmed that PM_2.5_ exposure led to significant inflammatory response in pulmonary tissues of mice, as evidenced by the significantly up-regulated expression of TNF-α and IL-6, which were in accordance with previous reports [8]. TGFβ1 is the most recognized isoform involved in respiratory diseases, which is important to the pro-fibrotic switch, matrix production, suppression of lung epithelial cells’ proliferation, and is enhanced during pulmonary inflammation progress [42, 43]. TGFβ1 promotes the expression of MMPs, including MMP2 and MMP9, and enhances the components of the extracellular matrix, such as collagens and fibronectins through up-regulating EMT-related transcription factors [32–34, 44]. A large number of studies have demonstrated the favorable effects of TGFβ1 on the pathological symptoms of lung injury, including PM_2.5_-triggered pulmonary damage, which is associated with the extracellular matrix (ECM) accumulation and fibrosis formation [45, 46]. In our research, we also demonstrated that PM_2.5_ exposure led to fibrosis in lung tissues, accompanied with significantly up-regulated expression of TGFβ1, MMP2, MMP9, Fibronectin, α-SMA and Vimentin.

Accumulating studies have indicated that TGFβ1 expression within the tumor microenvironment is frequently enhanced in NSCLC [47]. TGFβ1 stimulates tumor cell EMT, and promotes cell motility and metastasis via the activation of its down-streaming signaling pathways, subsequently favoring the expression of several transcriptional factors such as MMP2s, α-SMA and Fibronectin [48]. Therefore, TGF-β1 plays a critical role in inducing carcinogenesis. Previous studies have demonstrated that PM_2.5_ exposure could enhance NSCLC progression, which was partly associated with the EMT event [17, 49]. Given the considerably activation of TGFβ1 signaling during lung cancer progression, we hypothesized that NSCLC might be also associated with the long-term PM_2.5_ exposure. Herein, NSCLC biomarkers were measured in our study. RT-qPCR and IHC staining assays here demonstrated that the expression levels of lung cancer markers and cancer stem cell features of lung cells, including Kras, c-Myc, ABCG2, OCT4, SOX2 and Aldh1a1 [18, 36–38], were markedly increased in lung tissues of PM_2.5_-challenged mice. In contrast, tumor suppressors p53 and PTEN [39] were decreased in pulmonary samples of mice in response to PM_2.5_. All these *in vivo* findings validated that chronic PM_2.5_ exposure enhanced the risk of lung cancer.

Th17 cells are enriched within peripheral blood mononuclear cells and tumor tissues. IL-17a is mainly produced by Th17 cells, which is closely associated with the development of various types of tumors, including lung cancer [26–28, 50]. Elevated IL-17-producing cells were involved in the poor survival and lymphangiogenesis in NSCLC patients [51]. In addition, IL-17 up-regulated the net angiogenic activity and the *in vivo* growth of NSCLC through increasing CXCR2-dependent angiogenesis [52]. Here in our study, the markedly increased expression of IL-17a was detected in lung tissues, peripheral blood lymphocytes and splenic lymphocytes of mice with long-term PM_2.5_ exposure, further demonstrating the potential of PM_2.5_ in inducing lung cancer by increasing IL-17a expression levels. The clinical analysis in our study showed that IL-17a expression was markedly up-regulated in NSCLC patients, and its elevation was associated with the worse overall survival rate, which was in line with previous study [51]. Increasing studies have illustrated that Th17/IL-17a axis may promote the progression of solid tumors (lung, liver and ovarian cancers) and hematologic cancers (chronic lymphocytic leukemia and myeloma) [31, 53–56]. Here, IL-17a expression was greatly increased by PM_2.5_ in Th17 cells. NSCLC cells cultured in the conditional medium from PM_2.5_-incubated Th17 cells showed significantly elevated cell proliferation and EMT process compared to the control group. Functions of IL-17 relevant to tumor involve the induction of cytokines such as IL-6 and TGF-β1 [57, 58]. Consistently, our *in vitro* findings indicated that IL-17a treatment evidently up-regulated TGF-β1 expression, as well as its down-streaming signals such as MMP2, MMP9, α-SMA, Fibronectin and Vimentin, implying the EMT event in NSCLC cells. These *in vitro* results revealed the critical role of IL-17a induced by PM_2.5_ in promoting lung cancer development through accelerating EMT event. PM_2.5_-challenged mice with IL-17a knockout exhibited markedly alleviated lung injury and cancer stem cell property. In addition, the *in vivo* subcutaneous tumor model confirmed that A549-drived tumor growth was further accelerated by PM_2.5_, which was clearly abolished by the loss of IL-17a, accompanied with significantly reduced lung metastasis. Therefore, IL-17a might play a promotional role in PM_2.5_-induced progression of lung cancer.

In conclusion, our study for the first time provided solid evidence that long-term PM_2.5_ exposure could enhance the risk of NSCLC through promoting the expression of IL-17a, thereby increasing lung cancer cell proliferation and metastasis. Reducing IL-17a might be a promising strategy to treat air pollution-associated NSCLC. However, further studies are still warranted in future to investigate if there are other underlying mechanisms involved in PM_2.5_-promoted lung cancer development.

## MATERIALS AND METHODS

### PM_2.5_ sampling preparation

The procedures for PM_2.5_ sampling preparation were referred to previous study with certain modification [59, 60]. In brief, to collect exposure mass, quartz filter (8 cm × 10 cm, 2500QAT-UP, Pallflex Products, Putnam, USA) was used to continuously and weekly collect PM_2.5_ from Beijing Worker’s Sports Complex located in the central area of Beijing (Beijing, China) from January 2017 to June 2017 at a flow rate of 180 L/min. All particle size we collected was less than 2.5 μm. Ambient PM_2.5_ filters were stored at -80°C until experiments. The sampling was then treated with anhydrous alcohol and dissolved in pyrogen-free water. Subsequently, the extraction was sonicated for 2 days in an ultrasonic box and then concentrated through vacuum freeze-drying. Next, the double-distilled water was added to freeze-dried product, followed by centrifugation at 5000 rpm. The water-insoluble fraction was suspended in D-Hank’s buffer (Gibco Corporation, USA) for further experiments. The organic content has been added in the supporting files (Supplementary Table 1).

### Animals and treatments

Animal studies were performed in accordance with the principles of laboratory animal care (NIH publication revised 1985) and were approval by the Shandong Cancer Hospital and Institute, Shandong First Medical University and Shandong Academy of Medical Sciences (Ji’nan, China). The wild type, male C57BL/6J (IL-17a^+/+^) at 6-8 weeks of age were purchased from Beijing Vital River Laboratories (Beijing, China). The male IL-17a knockout (IL-17a^–/–^) mice (with C57BL/6J background) also at 6-8 weeks of age were purchased from the Jackson Laboratory (Bar Harbor, ME). Prior to the animal experiments, all mice were allowed to acclimation for the lab condition for a week before PM exposure (animal numbers were listed in the figure legends). They were raised in a specific pathogen-free (SPF) facility at a controlled temperature and humidity (25 ± 2°C, 50 ± 5% humidity) environment with a standard 12 h/12 h light/dark cycle. All mice were given *ad libitum* access to water and food in their cages.

As for PM_2.5_ challenge, IL-17a^+/+^ and IL-17a^-/-^ mice were exposed to either filtered air (FA) or PM_2.5_ (168.5 ± 4.9 μg/m^3^, flow rate of 80 L/min) for 6 h/day, 7 days/week for 1 week, 1 month or 3 months in an environmentally relevant and real-world PM with a PM-exposure system (Huironghe’s Air Pollution Exposure System for the Whole Body of Animals, HAPES, Beijing Huironghe Technology Co. LTD., Beijing, China) to perform whole-body exposure of mice. The control (FA) mice were exposed to an identical protocol with the exception of a high-efficiency particulate-air filter positioned in the inlet valve to remove all of the PM_2.5_ in the filtered air stream. After each treatment, all mice were sacrificed. Blood was collected from mice by cardiac puncture, and serum was centrifuged at 10,000 rpm for 10 min at 4°C. Then, the supernatant was collected for further analysis. The lung tissues were removed for histological, RT-qPCR and western blot analysis.

As for *in vivo* subcutaneous tumor model, the male C57BL/6 mice (8-10 weeks, IL-17a^+/+^ and IL-17a^-/-^) were used in the study. Hair in the lower dorsal skin of anesthetized mice was carefully trimmed with an electric clipper. A549 cells (1 × 10^6^ cells/mouse) in 100 μL of PBS were subcutaneously injected in the hair-trimmed area on day 0. Then, all mice were randomly divided into 5 groups (animal numbers were listed in the figure legends), and treated as followings: 1) the IL-17a^+/+^ Control (Ctrl) group without any treatments; 2) PM_2.5_-exposed group of IL-17a^+/+^ mice performed as described in the 4.2.1 section; 3) mice in the i.v. IL-17a group were injected with Recombinant Mouse IL-17a (1 μg/mouse per day; R&D System, USA) in sterile PBS intravenously through the tail vein; 4) PM_2.5_-exposed group of IL-17a^-/-^ mice also performed as described in the 4.21.1 section; 5) PM_2.5_-exposed group of IL-17a^-/-^ mice with the i.v. injection of Recombinant Mouse IL-17a (1 μg/mouse per day). During the treatments, the tumor size was measured every 2 days using calipers, and the body weight of mice was recorded. Tumor volume was calculated using the formula volume = (L × W^2^) × 0.52, where L is the largest diameter and W is the smallest diameter. After treatments for 4 weeks, all mice were sacrificed and the tumors were dissected for weighing and further analysis.

For tumor metastasis assay, the A549 cells (1 × 10^6^ cells/mouse) were implanted into IL-17a^+/+^ or IL-17a^-/-^ mice by tail vein injection. All mice were randomly divided into 5 groups (animal numbers were listed in the figure legends), and treated as followings: 1) the IL-17a^+/+^ Control (Ctrl) group without any treatments; 2) PM_2.5_-exposed group of IL-17a^+/+^ mice performed as described in the 4.2.1 section; 3) mice in the i.v. IL-17a group were injected with Recombinant Mouse IL-17a (1 μg/mouse per day; R&D System) in sterile PBS intravenously through the tail vein; 4) PM_2.5_-exposed group of IL-17a^-/-^ mice also performed as described in the 4.21.1 section; 5) PM_2.5_-exposed group of IL-17a^-/-^ mice with the i.v. injection of Recombinant Mouse IL-17a (1 μg/mouse per day). Tumor metastasis was monitored. At 60 days after injection, all mice were sacrificed, the tumor nodules formed on the lung surfaces were analyzed, and the lung tissues were embedded in paraffin for H&E staining or stored for RT-qPCR analysis.

### BALF isolation and analysis

BALF samples from each group of mice were simultaneously harvested. A total of 0.5 mL of BALF was collected. Then, total cells and neutrophils were counted using a hemocytometer in a double-blind manner for the calculation of the proportion of polymorphonuclear neutrophils. Protein concentrations in BALF were assessed using a BCA Protein Assay Kit (Pierce, USA) in accordance with the manufacturer’s protocols.

### ELISA assessments

TNF-α and IL-6 levels in serum (#ADI-900-047 for TNF-α, Peprotech, USA; #M6000B for IL-6, R&D System, USA) or supernatants (#DTA00D for TNF-α, R&D System; #D6050 for IL-6, R&D System) were measured using commercial kits according to the manufacturer’s instructions. IL-17a contents in BALF, serum or supernatants of mice were measured using ELISA kit (BEK-2054-2P, Biosensis, Australia) purchased from Amyjet Scientific (Wuhan, China) following the protocols recommended by the manufacturer.

### RT-qPCR

Total RNA was isolated from frozen lung tissues, tumor samples or the cultured cells using TRIZOL reagent (Invitrogen, USA) following the manufacturer’s instructions. Then, 2 μg of RNA was reverse-transcribed using the Transcriptor First Strand cDNA Synthesis Kit (Roche, USA). RT-qPCR assays were conducted by the use of the SYBR Premix Ex Taq II (TaKaRa, Dalian, China) on an ABI7900 real-time system (Applied Biosystems, USA). The PCR conditions were 95°C for 10 min; 40 cycles of 95°C for 10 s, 60°C for 10 s and 72°C for 20 s; and a final extension at 72°C for 10 min. The 2^–ΔΔCt^ method was used to quantify the relative expression level of RNA between groups. The transcription of the principal gene GAPDH was served as the internal control. The primer sequences for the genes used in the study were listed in Supplementary Table 2.

### Western blotting

Proteins were extracted from lung tissues, tumor samples or cultured cells and homogenized in lysis buffer (Beyotime, Nanjing, China). The protein concentrations were calculated using a BCA Protein Assay Kit (Pierce) following the instructions provided by the manufacturer. Protein samples (20-50 μg) were separated on 8-12% SDS-PAGE gels and transferred to polyvinylidene difluoride (PVDF) membranes (Millipore, MA, USA). The membranes were then blocked using Tris-buffered saline containing Tween-20 (TBST) with 5% skim milk powder for 1.5 h at room temperature, followed by incubation with primary antibodies (IL-17a, PA5-46947, ThermoFisher Scientific, USA; GAPDH, ab8245, Abcam) overnight at 4°C. Then, the membranes were incubated with a horseradish peroxidase (HRP)-conjugated anti-mouse or anti-rabbit IgG antibody (Abcam) for 1 h at room temperature. Membranes were finally treated with ECL reagents (Millipore) according to the manufacturer’s instructions. GAPDH was served as the loading control.

### Immunohistochemistry analysis

The pulmonary tissues or tumor samples were isolated from each group of mice as indicated, immersed in 4% paraformaldehyde for 24 h and then transferred to 70% ethanol. After dehydrated, all tissue samples were embedded in paraffin, and sectioned at 5-μm thickness. Then, the sections were stained with hematoxylin and eosin (H&E) or Masson’s trichrome kit (Nanjing Jiancheng Co., Ltd., Nanjing, China) according to the manufacturers’ protocols. Immunohistochemistry (IHC) was performed using the streptavidin peroxidase (SP, Jackson ImmunoResearch Inc., USA) method according to the kit’s protocols. The obtained tissue sections were then immersed in sodium citrate, heated in water-bath for 10 min at 98°C for antigen retrieval, and cooled to room temperature. Next, endogenous peroxidase was blocked using 3% hydrogen peroxide for 10 min. After washing with PBS, the tissue sections were treated with 5% normal goat serum (KeyGen Biotech, Nanjing, China), and then were incubated with the primary antibodies against c-Myc (ab32072, Abcam), OCT4 (ab18976, Abcam), SOX2 (PA1-094, ThermoFisher Scientific), IL-17a (ab79056, Abcam), IL-17a (PA5-79470, ThermoFisher Scientific) and KI-67 (MA5-14520, ThermoFisher Scientific) overnight at 4°C. Then, horseradish peroxidase (HRP)-conjugated secondary antibodies (Abcam) were used to visualize the antibody signal with diaminobenzidine (DAB, KeyGen Biotech). The tissue sections were calculated under a light microscope (Nikon, Japan). The quantification of IHC results was performed using Image-Pro Plus 6.0 software (Media Cybernetics, USA).

### Human samples

48 pairs of fresh lung cancer and the adjacent noncancerous tissues were collected from the Shandong Cancer Hospital and Institute, Shandong First Medical University and Shandong Academy of Medical Sciences (Ji’nan, China). The clinic pathological characteristics of patients were shown in Supplementary Table 3. These clinical tissue samples were histologically confirmed by H&E staining. The study was approved by the Ethics Committee of the Shandong Cancer Hospital and Institute, Shandong First Medical University and Shandong Academy of Medical Sciences, and was conducted in accordance with the ethical principles indicted by the Helsinki Declaration. The informed consent was obtained from all patients involved.

### Cells and culture

### Lymphocyte isolation from spleen

The spleen samples were isolated from each group of mice following PM_2.5_ challenge or not, and washed with PBS. Then, the spleen tissues were placed in a 200-mesh stain steel sieve over a culture dish supplemented with 5 mL lymphocyte isolation separation medium (Absin, Shanghai, China) and grounded into small pieces using the plunger of glass syringe. Then, the liquid was transferred into a centrifuge tube, and 200-500 μL of RPMI-1640 medium (Gibco) was added to the tube, followed by centrifugation at 800 × g for 30 min at room temperature. After centrifugation, three layers were formed. The middle milky layer containing lymphocytes was subsequently transferred into a new test tube. The obtained lymphocytes were rinsed with PBS, suspended in RPMI-1640 medium (Gibco) with 10 % fetal calf serum (Solarbio), and then transferred into a culture bottle. All these procedures were performed under sterile condition. Finally, the lymphocyte viability was measured following the trypan blue exclusion criteria, and the viability was over 95%. The purified lymphocyte was finally used for ELISA analysis.

### Peripheral blood mononuclear cells (PBMCs) isolation

PBMCs were isolated from mouse peripheral blood samples using the standard Ficoll-Hypaque density gradient centrifugation methods as previously indicated [61, 62]. In brief, T lymphocytes were prepared from mouse PBMCs by negative selection with magnetic bead depletion of non-T lymphocytes with the EasySep mouse T lymphocytes isolation kit (Stemcell Technologies, USA) according to the instructions provided by the manufacturer. Then, the purity of T lymphocytes was calculated using the flow cytometry analysis and was found to be greater than 95%.

### Th17 differentiation, NSCLC cell culture and treatment

At first, total splenic T cells were purified using the negative selection with the EasySep™ Mouse CD4^+^ T Cell Isolation Kit (Stemcell Technologies, Canada) according to the manufacturer’s protocols, and then were purified to > 95%. CD4^+^ purity was assessed through FACS-sorting using anti-CD4 FITC (Becton Dickinson, USA). For gating, viable cells were selected according to their Forward-scatter (FSC) values. Next, T-helper cells were isolated as CD4^+^ cells (> 97% total viable cells). T cells were planted in 12-well plates supplemented with plate bound 5 μg/ml of anti-CD3 (Abcam) and 2 μg/ml of soluble anti-CD28 (Abcam) antibodies at 2 × 10^6^ cells/well in RPMI 1640 medium (Gibco) containing 15% inactivated fetal bovine serum (FBS, Gibco). For Th17 cell differentiation, splenic T cells were incubated with IL-6 (20 ng/ml), IL-23 (15 ng/ml), IL-1β (10 ng/ml), and TGF-β (5 ng/ml) (all from Invitrogen, USA), and IFN-γ-IL-2- and IL-4 directed antibodies (R&D Systems) as previously indicated [63]. Th17 differentiation was measured using flow cytometry [64]. Then, PM_2.5_ at 100 μg/cm^2^ was subjected to the obtained Th17 cells for 24 h. The supernatants and cells after treatments were collected for ELISA, RT-qPCR, western blot and IF assays.

Human NSCLC cells including A549 and H1350 were purchased from the American Type Culture Collection (ATCC, Manassas, USA). All these cancer cells were grown in RPMI 1640 medium (Gibco, USA) supplemented with 10% FBS (Gibco) and 100 U/mL penicillin/streptomycin. Cells were grown at 37°C in a humidified atmosphere containing 5% CO_2_ and 95% air. Recombinant human IL-17a (R&D System) was used to stimulate NSCLC cells as described in the figure legends.

### CCK-8 analysis and EdU determination

After each treatment, the cell viability analysis was conducted using the CCK-8 detection kit (Dojindo, Kumamoto, Japan) according to the protocols as described by the manufacturer. Finally, the absorbance was measured with a microplate reader at a wavelength of 450 nm.

After treatments, the cell proliferation ability was calculated through the EdU incorporation assay with the EdU Assay Kits (Life Technologies, USA) following the protocols recommended by the manufacturer, and was finally observed with a fluorescent microscope (Olympus, Japan).

### Colony formation analysis

For colony formation analysis, 1000 cells after treatments were plated in a 10 cm dish and allowed to grow for 14 days at 37°C in 5% CO_2_. Then, the surviving colonies (more than 50 cells in each colony) were counted using a microscope following Giemsa staining.

### Immunofluorescence (IF) staining

After treatments, the cells were fixed in 4% paraformaldehyde for 20 min and permeated using 0.5% Triton X-100 (Solarbio, Beijing, China) in PBS for 5 min. After blocking with 5% goat serum (Solarbio) for 1 h, the cells were incubated with primary antibodies (anti-IL-17a antibody, Abcam, ab79056; anti-N-cadherin antibody, Abcam, ab18203) at 1:100 dilutions overnight at 4°C in a humidified chamber, followed by incubation with fluorescently labeled secondary antibodies (goat anti-rabbit IgG H&L, Abcam, ab150077; goat anti-rabbit IgG H&L, Abcam, ab150088) for 1 h. Then, the cells were counterstained with DAPI (Beyotime) for nuclear staining. The images were observed under a fluorescence microscope (Olympus), and the images were analyzed using Image Pro Plus 6.0.

### Transwell analysis

For the migration analysis, the treated cells were suspended in serum-free DMEM (Gibco), and 1 × 10^5^ cells were then plated in the top chamber lined with an uncoated 8.0 μm pore membrane (Millipore). For the invasion analysis, 1 × 10^5^ cells were plated in the top chamber coated with Matrigel (Corning, USA). Subsequently, the chambers were inserted into a 24-well plate supplemented with DMEM containing 20% FBS (Gibco). After incubation for 24 h at 37°C in 5% CO_2_, the cells undergoing migration or invasion through the membrane were stained using 0.1% crystal violet, and were quantified with a microscope (Olympus).

### Wound healing assays

Cell motility was calculated through the use of a scratch wound assay. The cells and the controls were incubated in 6-well dishes with or without the conditional medium until 80-90% confluent. Then, the cell layers were carefully wounded with sterile tips and washed twice with fresh medium. Cells were incubated with fresh medium and observed under a microscope at 24 h after wounding. Three random visual fields were quantified.

### Statistical analysis

Data in our study were represented as Means ± standard error of the mean (SEM). Differences among groups were analyzed using one-way analysis of variance (ANOVA), followed by a post hoc Tukey’s test. Comparisons between two groups were performed using an unpaired Student’s t-test. The survival rate was analyzed using the Kaplan-Meier method. All results were analyzed using GraphPad Prism Software Version 6.0 (GraphPad Software, California, USA). Differences were considered to be significant when p value < 0.05. All experimenters were blinded to the animal genotype and grouping information. Data from the animal experiments were collected through a blinded manner. All *in vitro* experiments were repeated at least three independent times unless specifically demonstrated in the figure legends.

## Supplementary Material

Supplementary Tables
