# Supplementary material for: Long-term PM2.5 exposure increases the risk of non-small cell lung cancer (NSCLC) progression by enhancing interleukin-17a (IL-17a)-regulated proliferation and metastasis
Source: Aging (Albany NY). 2020 Jun 18;12(12):11579–602. doi: 10.18632/aging.103319 (PMC7343463; doi:10.18632/aging.103319)
Supplement: Supplementary Tables [file aging-12-103319-s001..pdf]

SUPPLEMENTARY TABLES

Supplementary Table 1. Components of the collected $PM_{2.5}$ (ng/m^3) used in the study.

Element	Ambient $PM_{2.5}$
Magnesium	26.75 ± 3.96
Selenium	0.46 ±0.04
Cerium	0.04 ± 0.01
Copper	2.96 ± 0.32
Potassium	36.74 ± 7.58
Barium	3.29 ± 0.18
Sodium	59.07 ± 8.23
Lead	2.08 ± 0.96
Sulfur	1036.72 ± 378.29
Aluminum	28.63 ± 8.47
Cadmium	0.53 ± 0.07
Strontium	0.48 ± 0.18
Rubidium	0.08 ± 0.02
Nitrogen	0.39 ± 0.28
Cobalt	0.11 ±0.01
Iron	59.25 ± 11.29
Lanthanum	0.02 ± 0.01
Manganese	1.72 ± 0.47
Antimony	0.52 ± 0.29
Molybdenum	0.74 ± 0.20
Chromium	7.19 ± 1.08
Vanadium	0.36 ± 0.14
Phosphorus	17.07 ± 3.08
Arsenic	0.92 ± 0.18
Titanium	0.97 ± 0.05
Calcium	93.53 ± 14.09
Zinc	10.96 ± 1.68

Supplementary Table 2. The primer sequences for the genes used in the study.

Primers	Forward Sequence (5'-3')	Reverse Sequence (5'-3')
mMMP2	TTCCGCTTCCAGGGCACA	CACCTTCTGAGTTCCCACCAA
mTNF-α	ACCTGGCCTCTCTACCTTGT	CCCGTAGGGCGATTACAGTC
mIL-6	CAACGATGATGCACTTGCAGA	TCTCTCTGAAGGACTCTGGCT
mMMP9	CGTCGTGATCCCCACTTACT	CGTCGTGATCCCCACTTACT
mTGF-β1	GACTCTCCACCTGCAAGACC	GACTCTCCACCTGCAAGACC
mα-SMA	CTGCCGAGCGTGAGATTGT	CTTCGTCGTATTCCTGTTTGCT
mFibronectin	CATGAAGGGGGTCAGTCCTA	TAGGTTTGCAGGTCCATTCC
mVimentin	TGGACGTTTCCAAGCCTGAC	CTGTCTCCGGTACTCGTTTGACT
mKras	AGACACGAAACAGGCTCAGG	GCATCGTCAACACCCTGTCT
mc-Myc	TCACCAGCACAACTACGCCG	CAGGATGTAGGCGGTGGCTT
mABCG2	TCGCAGAAGGAGATGTGTTGAG	CCAGAATAGCATTAAGGCCAGG
mOCT4	AGCTGCTGAAGCAGAAGAGG	AGATGGTGGTCTGGCTGAAC
mSOX2	GCGGAGTGGAAACTTTTGTCC	CGGGAAGCGTGTACTTATCCTT
mAldh1a1	ATGGTTTAGCAGCAGGACTCTTC	CCAGACATCTTGAATCCACCGAA
mp53	TAACAGTTCCTGCATGGGCCGC	AGGACAGGCACAAACACGCACC
mPTEN	AGACCATAACCCACCACAGC	AGTGCCACGGGTCTGTAATC
mIL-17	ACCGCAATGAAGACCCTGAT	CAGGATCTCTTGCTGGATGAGA
mGAPDH	GGTGAAGGTCGGTGTGAACG	CCCGTAGGGCGATTACAGTC
hIL-17	CTACAACCGATCCACCTCACC	AGCCCACGGACACCAGTATC
hMMP2	TGAGCTATGGACCTTGGGAGAA	CCATCGGCGTTCCCATAC
hMMP9	CATCGTCATCCAGTTTGGTG	AGGGACCACAACTCGTCATC
hTGF-β1	GTACCTGAACCCGTGTTGCT	GTATCGCCAGGAATTGTTGC
hα-SMA	CATGGCATCATCACCAACTG	GCTGGGACATTGAAAGTCTC
hFibronectin	AGATGAGTGGGAACGAATGTCT	GAGGGTCACACTTGAATTCTCC
hVimentin	GCTGAATGACCGCTTCGCCAACT	AGCTCCCGCATCTCCTCCTCGTA
hGAPDH	GGAGTCAACGGATTTGGTC	GGCAACAATATCCACTTTACC

Supplementary Table 3. Correlation between expression of IL-17a and clinical pathologic features of NSCLC patients.

Characteristic	IL-17a		*P*-values
	Low (n=74)	High (n=42)	
**Age**			
≥50	43	23	0.426
<50	31	19	
**Gender**			
Male	49	28	0.547
Female	25	14	
**Pathologic type**			
Squamous cell carcinoma	42	20	0.086
Adenocarcinoma	32	22	
**Tumor size (cm)**			
≥3	42	18	0.132
<3	32	24	
**Tumor location**			
Left lung	36	22	0.359
Right lung	38	20	
**TNM classification**			
T1	14	10	0.028*
T2	31	15	
T3	20	9	
T4	9	8	
**Distant metastasis**			
No	53	16	0.031*
Yes	21	26	
